# A visual search asymmetry for relative novelty in the visual field based on sensory adaptation

**DOI:** 10.3758/s13414-019-01943-w

**Published:** 2019-12-24

**Authors:** Michael J. Morgan, Joshua A. Solomon

**Affiliations:** Centre for Applied Vision Research, School of Health Sciences, City, University of London, Northampton Square, London, EC1V 0HB UK

**Keywords:** Aftereffect, Popout, Change blindness

## Abstract

The ability to detect sudden changes in the environment is important for survival. However, studies of “change blindness” have shown that image differences are hard to detect when a time delay or a mask is imposed between the different images. However, when sensory adaptation is permitted by accurate fixation, we find that change detection is not only possible but asymmetrical: a single changed target amongst 15 unchanging distractors is much easier to detect than a target defined by its lack of change. Although adaptation may selectively reduce the apparent contrast of unchanged objects, the asymmetry in “change salience” cannot be attributed to any such reduction because genuine reductions in target contrast increase, rather than decrease, target detectability. Analogous results preclude attribution to apparent differences between (a) target onset and distractor onset and (b) their temporal frequencies (both flickered at 7.5 Hz, minimizing afterimages). Our results demonstrate a hitherto underappreciated (or unappreciated) advantage conferred by low-level sensory adaptation: it automatically elevates the salience of previously absent objects.

## Introduction

Change in a scene can be a potent method for identifying an object of possible interest. Before artificial computers, astronomers used to detect the presence of planets, comets, and asteroids by presenting two images in rapid succession, taken at the same sidereal time but on different dates. Any object that had moved against the background of the fixed stars would pop out because of its movement. Paradoxically, however, studies of “change blindness” (O’Regan, Rensink, & Clark, 1996; Rensink, O’Regan, & Clark, 1997; Wright, Alston, & Popple, 2001; Wright, Green, & Baker, 2000) have shown that the human visual system is severely limited in its ability to detect even large changes between scenes. These latter studies differ from the astronomical method in that they deliberately introduce a sufficiently long blank interval or mask between the two images to prevent low-level motion detection. It has thus become generally accepted that motion detection is the only useful mechanism available to the visual system for detecting uncued changes within complex images.

**Fig. 1:**
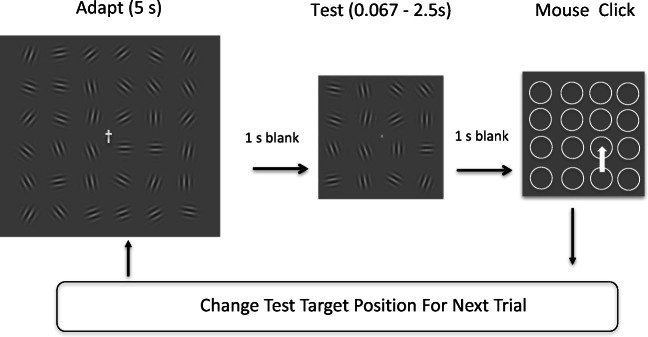
Baseline (target-change) conditions. The adapting stimulus on the left was exposed for 5 s while observers fixated on the central cross. Gabors flickered at a rate of 7.5 Hz to prevent the buildup of afterimages. During adaptation observers fixated the asymmetrical cross in the center and reported conjunctions of its contrast and shape. After adaptation, the test stimulus was presented for a variable duration (0.067 – 2.5 s), before being replaced by a set of placeholder circles. Observers selected one of these circles by clicking with a mouse. The correct circle, which was in the position of the unique test Gabor that had been rotated 90° from its corresponding adaptor, disappeared after the click. This provided observers with feedback, enabling them to learn the task. Following this feedback, a new trial was initiated, in which the adapting stimulus was the same but the target position was selected at random. In target-same conditions (not illustrated), all the Gabors in the test *except* the target were rotated 90° from their spatially corresponding adaptors

**Fig. 2:**
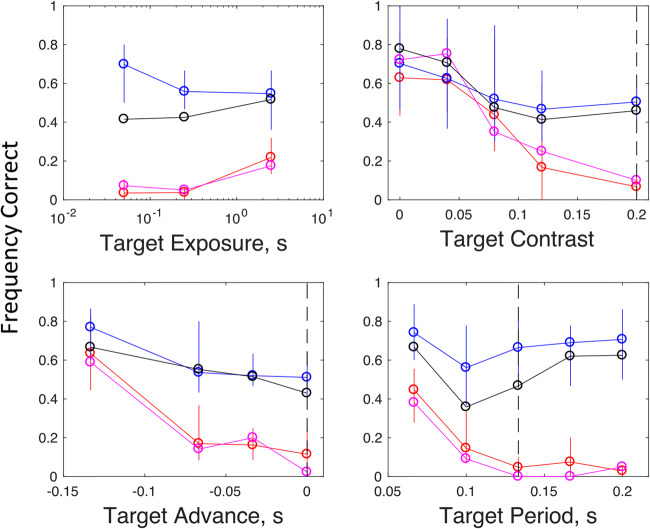
Frequency of correct detections plotted against various properties of the test stimulus in four separate experiments (one experiment per panel). Circular symbols and solid vertical lines show the mean and range (over N = 4 observers) in target-change (blue and black) and target-same (red and magenta) conditions. Baseline properties shared by all distractors are indicated by the dashed vertical lines. The black and magenta symbols illustrate results from the first trial in each block; blue and red symbols illustrate results from all trials

**Fig. 3:**
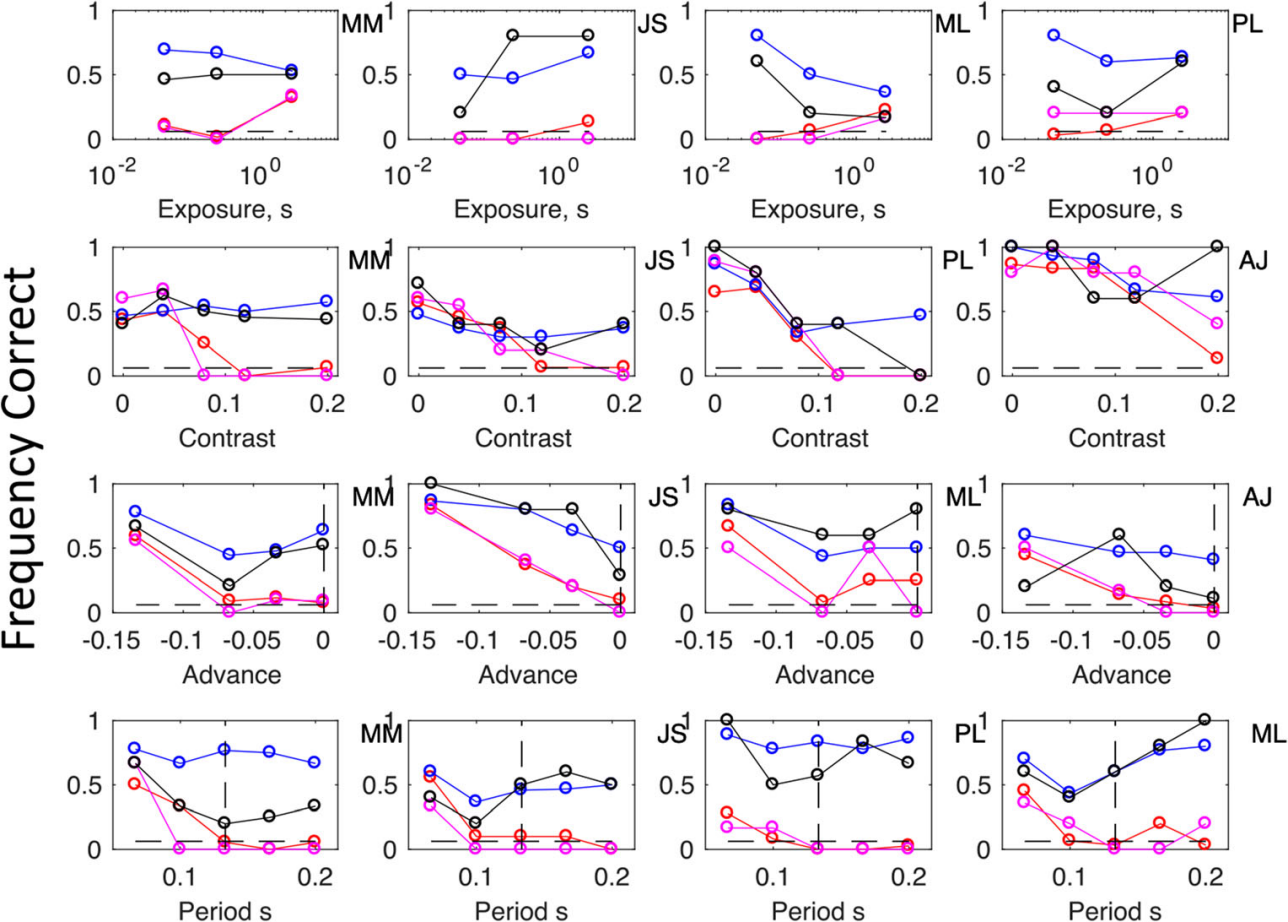
The figure shows results for individual observers (MM, JS, ML, AJ, PL) in the experiments summarized in Fig. 2, using the same conventions as in Fig. 2.

Recently, however, we showed that new objects can indeed pop out, even when motion detection is prevented by insertion of a blank interval (Morgan & Hauperich, 2016; Morgan & Solomon 2019). In the current report we contrast how easy it is to detect change with how difficult it is to detect the absence of change. Search asymmetries like this are thought to be indicative of primitive visual features (Treisman & Souther, 1985). We consider and reject the possibility that adaptation produced pop out by giving the target a uniquely high value of apparent contrast, an earlier apparent onset, or an apparently different temporal frequency. Instead, we conclude that adaptation facilitates detection by imbuing the target with a hitherto unrecognized, primitive visual feature: “relative novelty.”^1^

Figure 1 illustrates the “baseline” conditions, one of which was included in all of our experiments. Each trial began with 5 s of the adapting stimulus: a 6 × 6 array of flickering grating patches (Gabors). During adaptation, observers kept their gaze fixed on a central cross that changed its contrast or its shape (upright vs. inverted), or both, at a rate of 1.5 Hz. The observer had the task of pressing a key whenever there was a rare conjunction of contrast and shape (Schwartz, Vuilleumier, et al., 2005). The purpose of this central task was to distract observers and prevent them from remembering the orientations of the adapting Gabors. Adaptation was followed by a 1-s blank screen and then the test stimulus. In “target-change” conditions one of the Gabors was rotated 90° from its spatially corresponding adaptor. In the “target-same” conditions, all Gabors *except* the target were rotated 90°. Trials were blocked by condition. Participants were instructed to identify the odd-man-out in the test array by clicking on its position in the subsequent array of circles. Following their click, the actual target was indicated by the disappearance of its corresponding circle. After six trials the session terminated, and participants rested before the next session with a new adaptor array, in which the orientations of the Gabors were once again randomly selected, to prevent accumulation of adaptation over sessions.

In four separate experiments we investigated the effects of test duration, target contrast, target delay, and target temporal frequency on detection of the odd-man-out. The purpose of these investigations was to determine which, if any, of these stimulus attributes were critical for detecting change.

## Experiment 1: Detection of change with brief exposures

In Experiment 1 we varied the exposure duration of the test stimulus between 0.067 and 2.5 s. Results are shown in the top left panel in Fig. 2. Individual data are shown at the top of Fig. 3. When the odd-man-out was defined as the singleton that did *not* change, performance exceeded chance (1/16) only with the longest test duration (2.5 s), which allowed for detailed inspection of individual Gabors. Performances in target-change conditions actually decreased with test duration; a result inconsistent with any sort of serial search (Treisman & Gelade, 1980). Figures 2 and 3 also illustrate results from the first trial in each block. As previously reported by Morgan and Solomon (2019), first-trial performances are a bit worse than overall performances, but they remain significantly above chance in target-change conditions. This shows that stimulus change (i.e., between adaptor and test) is necessary for the task, since logically there is no other basis for detection on Trial 1. It also shows that the effect builds up over trials, which we take as evidence for gradual adaptation. Finally, we note that the asymmetry cannot be ascribed to dilution of adaptation by the test array, because it is present in the results from first-trial performances.

## Experiment 2: Effect of relative target contrast on detection

Using the shortest duration (0.67 s) only, Experiment 1 was repeated, except that the contrast of the target Gabor was reduced to 60%, 40%, 20%, and even 0% relative to the distractors. Experiment 1’s baseline condition was replicated by including targets having a contrast equal to (i.e., 100%) that of the non-target (i.e., distractor) Gabors.

Results (Fig. 2, top right) showed that reducing target contrast relative to the distractors progressively improved performance in target-change conditions and target-same conditions. For any given target contrast, average performance in the target-change condition was always at least as good as the average performance in the target-same condition. As no reduction in target contrast produced any reduction in performance, we reject the possibility that the target change was being detected only because it made the target appear to have higher contrast.

## Experiment 3: Effect of target delay

Next, we considered the possibility that adaptation to a stimulus introduces a subsequent delay in the neural response to a similar stimulus, as in the Pulfrich effect for contrast (Solomon & Morgan, 1996). In the baseline (target-change) condition this would make the distractors appear after the target; in target-same conditions, the target would appear only after a delay. To see if this could have been the cause of the asymmetry due to adaptation in Experiment 1, we repeated that experiment adding a delay to the target, to see if it would counteract the effect of adaptation. We used the medium exposure from Experiment 1 (0.25 s) so that the target and distractors overlapped in time. Contrary to the delay explanation, small delays had no effect on performance. Only with the largest of our delays (eight frames, 0.133 s) was there any change in performance. In this case, detection was facilitated in both conditions: target-change and target-same. Consequently, we can be confident that the visual search asymmetry for relatively novel targets is not due to their appearing before the previously seen distractors.

## Experiment 4: Effect of temporal frequency

As another potential explanation of the asymmetry, we considered speed. It is known that adaptation to a moving stimulus reduces the perceived speed of subsequent tests (Thompson, 1981), and further evidence suggests that slowly moving stimuli are harder to find amongst faster distractors than vice versa (Ivry & Cohen, 1992; Rosenholtz, 1999). These results are not directly relevant to our stimuli, which were flickering rather than moving, but it is possible that the previous results for speed are mediated by changes in perceived temporal frequency. To investigate whether the visual search asymmetry for our novel targets could be attributed to their having a uniquely high or low apparent frequency, we tried increasing and decreasing the temporal frequency of the target, while keeping the distractors’ frequency constant. We used the medium exposure from Experiment 1 (0.25 s) to restrict the bandwidth of the frequency. The results (Fig. 2, bottom right) showed that reducing the temporal frequency of the target facilitated its detection in the target-same conditions, but had little effect in the target-change conditions, suggesting that detection in the latter case was not based on apparent temporal frequency.

## Discussion

Our results contradict the widely held view that the visual system is poor at detecting change amongst multiple items in the absence of transients. It is generally agreed that even no-change detection is possible when transients are allowed (i.e., when adopting an inter-stimulus interval of zero; Theeuwes, 2004). However, many previous studies of change blindness (e.g. O’Regan et al., 1996; Rensink et al., 1997) did not ensure that the pre-change and post-change stimuli fell on the same part of the retina. The crucial importance of this was revealed in our previous (2019) paper, which showed that if the eyes moved between adaption and test, detection was possible only if the test also moved so as to fall on the same part of the retina as the previous adaptor. The fact that the adaptation is retinotopic is strong evidence for an early site of the adaptation, probably V1 (Kohn & Movshon, 2003).

Of those previous studies that did control fixation (Wright et al., 2000, 2001), the 150-ms exposure was presumably too brief to allow adaptation. The array also changed on every trial, again preventing any build-up of adaption. The only study of which we are aware where adaptation was allowed to build up is in an otherwise unpublished abstract (Rensink, 1996). It describes an experiment in which a visual texture was alternated with a blank field until the observer reported the presence of a target. The texture consisted of horizontal and vertical rectangles, and the target was either a rectangle that changed orientation between frames or the only rectangle not to change orientation. The latter condition was more difficult, echoing our finding of an asymmetry between change detection and no-change detection. Rensink’s explanation of the difference is based upon the assumption that observers memorized a subset of elements in the array, and that there was a different memory capacity in the two conditions. This explanation is not appropriate to our experiments, where memorization was discouraged by the attention-consuming central task.

The asymmetry we have found between change and no-change detection echoes that reported in Visual Working Memory (VWM) tasks, comprehensively reviewed by Hyun, Woodman, Vogel, Hollingworth, and Luck (2009). However, for the following reasons, we do not think that the ability to detect change in our task depends on VWM: (1) the capacity of VWM is limited to about four items (Luck & Vogel, 1997). Consequently, if our observers had been limited by VWM, their performances could not have exceeded about 25% (i.e., four out of 16) correct. In fact, all our observers were able to exceed 50% correct. (2) We used a central distracting task to prevent conscious attention to the adapting array, and the accuracy of working memory is known to decline when observers are prevented from attending to relevant positions in the visual field (Awh & Jonides, 2001). (3) Performance in our task depends on retinotopic adaptation (Morgan & Solomon 2019). VWM, on the other hand, is capable of comparing items presented to different regions of the retina.

We find that making the target different from the distractors in contrast, delay, or temporal frequency has little effect on detection of a target defined by relative novelty (i.e., change from its corresponding adaptor). However, these changes do improve detection of a target defined by being the same as its adaptor, when the distractors are different. Pop out in the latter case may have nothing to do with a novelty-based mechanism. It is based on the target’s atypicality, just as in traditional pop-out experiments with targets differing in color or orientation from otherwise uniform distractors.

Our results are in accord with many demonstrations of efficient change-detection in the auditory system, where the easier detection of a change versus no-change has also been found. For example, Sohoglu and Chait's (2016) participants were able to detect the onset of previously absent temporal frequencies with more ease than they were able to detect the cessation of previously repeated temporal frequencies. It seems plausible that the introduction of *any* previously absent stimulus is likely to be especially salient. We suggest that the difference between “change blindness” in vision and “change salience” in audition arises because auditory adaption does not depend upon a stationary head. By allowing retinotopic adaptation to occur in vision, we have bridged the gap between vision and audition.

Search asymmetries have been considered a litmus test for primitive visual features ever since Triesman and Souther (1985) reported that it was easier to find a Q amongst Os than it was to find an O amongst Qs. In this report we have shown that it is easier to find a changed target amongst unchanged distractors than *vice versa*. Furthermore, we have shown that our changed targets did not contain higher apparent contrast nor different apparent frequency nor any onset asynchrony compared to its distractors. Consequently, we conclude that (relative) novelty itself is its primitive visual feature.

## Methods

### Apparatus and subjects

Stimuli were presented on a 60-Hz frame-rate Sony Trinitron monitor in a darkened room, viewed from 0.75 m, so that one pixel subtended 1.275 arcmin at the observer’s eye. Viewing was binocular through natural pupils, with observers wearing their normal correcting lens for the viewing distance if necessary. A total of five observers participated in the experiments, comprising the two authors and three other experienced psychophysical observers from City, University of London, who were naïve as to the purpose of the experiment. Note that we report only within-observer statistics. Our five observers’ performances need not and should not be considered representative of the population at large.

### Stimuli

The stimuli (e.g., Fig. 1) consisted of rectangular arrays of Gabors, each of which comprised a sinusoidal grating of spatial frequency 3.75 cyc/deg multiplied by a circular Gaussian envelope. The grating shifted phase by π/8 radians every video frame giving it a temporal frequency of 7.5 Hz. The Gaussian envelope had a spread (*σ*) of 0.21°. The mean luminance and contrasts of the Gabors were 70 cd/m^2^ and 0.2, respectively. The envelope did not move. The envelope was truncated at ±3*σ*. The adapting array comprised 6 × 6 randomly oriented, equally spaced Gabors, with a center-to-center spacing of 1.87°. The test array consisted of a 4 × 4 array in the positions of the central 4 × 4 elements of the previous adapting array. The purpose of this was to avoid possible edge effects at the boundaries of the adapting array.

### Procedure

Adaptation was produced by presenting one of these Gabor arrays for an initial 5 s, during which the observer was instructed to fixate a stationary point in the center of the display, and to carry out a task based on additional stimuli presented there. The first adaptation period was followed, after 1 s, by a test. Change was introduced by rotating one or more of the gratings 90° from its adapting orientation. After the test, the stimuli were replaced by a set of circular placeholders, and the observer used a mouse to click on the position of the target. To give feedback, the target’s placeholder was switched off to show the target’s position after the mouse click. After the mouse click, the screen went blank while the next set of Gabors was calculated (approx. 1 s) and then the next adapting stimulus was presented.

Each session consisted of six trials in one condition. Experiments 1, 2, 3, and 4, had 6, 10, 8, and 10 conditions, respectively (see Figs. 2 and 3). Half of all conditions were “target-change”; the other half were “target-same.” Subjects were encouraged to take a 5-min rest outside the experimental room between sessions. The number of sessions for each observer depended to some extent on availability, and some conditions had more sessions than others. For Observer MM in Experiment 1 (top row, left panel in Fig. 3), the minimum number of sessions was 16. For JS, ML, and PL (i.e., continuing to read left to right at the top of Fig. 3), it was 7, 7, and 5, respectively. For the remaining experiments (lower three rows), reading Fig. 3 from left to right, the minimum numbers of sessions were Experiment 2: 7, 6, 6, 5; Experiment 3: 15, 5, 3, 6; and Experiment 4: 3, 5, 6, 6.

### The central task for distracting attention

To take attention away from the adapting stimulus during adaptation, and to discourage active memorization of the stimuli, observers carried out a demanding task, based on stimuli appearing at fixation. In the center of the adapting array, superimposed on the white fixation point, a series of asymmetrical crosses were presented at a frequency of 1.5 Hz, allowing seven crosses per adapt period. The crosses were either upright or inverted, and could be high-contrast white, high-contrast black, low-contrast white, or low-contrast white. (We chose contrast rather than color as a cue because one of the observers was color anomalous.) Most upright crosses were either high-contrast white or low-contrast black. The observer’s task was to press a button when there was a rare conjunction of contrast and orientation, for example, a low-contrast white, upright cross. Observers were instructed to press a key as soon as they saw one of these rare conjunctions. The observers were encouraged to avoid false positives. In practice, false-positive rates were very low (0.0148 over all conditions), therefore, no measures were taken to eliminate trials on which they occurred.

## Footnotes

1 We must contrast relative novelty, which is inherent in previously absent stimuli, with the novelty of never-before-seen stimuli. The latter stimuli do not pop out from distractors composed of stimuli that merely have not been seen recently (e.g., Wolfe & Horowitz, 2004). Relative novelty is defined with respect to the relatively recent past and is spatially local. If a given stimulus recently appeared elsewhere, it can still be relatively novel at other positions within an image.
